# Time-of-Day Control Double-Order Optimization of Traffic Safety and Data-Driven Intersections

**DOI:** 10.3390/ijerph16050870

**Published:** 2019-03-09

**Authors:** Chen Xu, Decun Dong, Dongxiu Ou, Changxi Ma

**Affiliations:** 1 The Key Laboratory of Road and Traffic Engineering, Ministry of Education, School of Transportation Engineering, Tongji University, Shanghai 201804, China; 1510706@tongji.edu.cn (C.X.); ddc@tongji.edu.cn (D.D.); ou.dongxiu@tongji.edu.cn (D.O.); 2 School of Traffic and Transportation, Lanzhou Jiaotong University, Lanzhou 730070, China

**Keywords:** traffic safety, time-of-day control, double-order optimization, data-driven

## Abstract

This paper proposes a novel two-order optimization model of the division of time-of-day control segmented points of road intersection to address the limitations of the randomness of artificial experience, avoid the complex multi-factor division calculation, and optimize the traditional model over traffic safety and data-driven methods. For the first-order optimization—that is, deep optimization of the model input data—we first increase the dimension of traditional traffic flow data by data-driven and traffic safety methods, and develop a vector quantity to represent the size, direction, and time frequency with conflict point traffic of the total traffic flow at a certain intersection for a period by introducing a 3D vector of intersection traffic flow. Then, a time-series segmentation algorithm is used to recurse the distance amongst adjacent vectors to obtain the initial scheme of segmented points, and the segmentation points are finally divided by the combination of the preliminary scheme. For the second-order optimization—that is, model adaptability analysis—the traffic flow data at intersections are subjected to standardised processing by five-number summary. The different traffic flow characteristics of the intersection are categorised by the K central point clustering algorithm of big data, and an applicability analysis of each type of intersection is conducted by using an innovated piecewise point division model. The actual traffic flow data of 155 intersections in Yuecheng District, Shaoxing, China, in 2016 are tested. Four types of intersections in the tested range are evaluated separately by the innovated piecewise point division model and the traditional total flow segmentation model on the basis of Synchro 7 simulation software. It is shown that when the innovated double-order optimization model is used in the intersection according to the ‘hump-type’ traffic flow characteristic, its control is more accurate and efficient than that of the traditional total flow segmentation model. The total delay time is reduced by approximately 5.6%. In particular, the delay time in the near-peak-flow buffer period is significantly reduced by approximately 17%. At the same time, the traffic accident rate has also dropped significantly, effectively improving traffic safety at intersections.

## 1. Introduction

Traffic control is an efficient way to reduce accidents at intersections. In major cities in China, the main intersections are easily blocked, especially when the traffic is close to saturation at the intersection peak. The control strategy of vehicle-actuated control is prone to conflict and chaos, which easily results in paralysis at intersections, an overflow of traffic, or even major congestion in the main trunk road or in an entire area. At this point, the traffic manager’s goal of control system safety and stability is often significantly greater than the advancement. The analysis from the aspects of traffic safety and implementation cost indicates that time-of-day control remains the mainstream control method used by most cities [1,2,3,4]. 

Time-of-day control (TOD) involves dividing 24 h into several time periods according to the change in flow at intersections and using different signal control schemes for different traffic time periods. The traffic signal controller is programmed to switch automatically according to the scheduled time division scheme. The time-of-day control has low dependence on the collection of traffic information and is reliable. Studies have shown that time-of-day control that is highly matched with traffic flow can significantly improve traffic discharge capacity and effectively reduce traffic delay.

**Figure 1:**
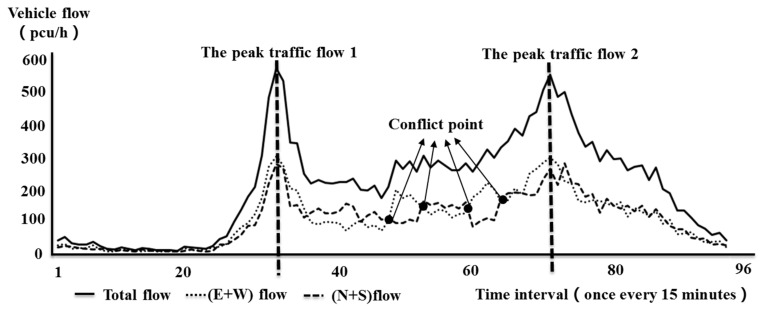
Distribution chart of total and fractional flows of each direction (intersection of Yangming North Road and Renmin East Road, Yuecheng District, Shaoxing City on 23 June 2016).

The current traditional mainstream segmented point division model considers the traffic flow in an entire day at intersections as the major judgment factor, and turning movement counts and other factors are taken as auxiliary evidence. The clustering algorithm is used to recurse the time of high correlation, and the boundary point is the time-of-day control segmented point. Some problems are determined after in-depth research into the above traditional segmented point division model. The traffic flow data at the intersections of Yangming North Road (north–south) and Renmin East Road (west–east) of Yuecheng District, Shaoxing, China, on 23 June 2016 are taken as examples in Figure 1.

In Figure 1, the solid line represents the total flow rate of the intersection of four inlet flows. The large dashed line represents the combined flow of the north and south directions and the flow rate of the north inlet. The dotted line represents the east–west inlet flow and the sum of west inlet flow. Data are collected every 15 min, and 96 datasets are collected throughout the day. Figure 1 shows that the peak traffic flow between peaks 1 and 2 is generally stable, and, if the traditional method of total traffic intersection division is followed, this level should be divided into one control period. However, when conducting data analysis and data mining on west–east and north–south traffic flow under the total flow, it is found that during the normal period of traffic flux, the east-west flow and the north-south flow generate several crossing points, which are defined as conflict points and shown as black dots in Figure 1. The emergence of this conflict point indicates that the total flow rate of the intersection is stable. Its north–south direction and east–west flow are interleaved in the general direction, and the total flow direction exhibits a fundamental change. In the first half, a large flow exists in the north and south. In the second half, a large flow occurs in the east–west direction, and the change in internal traffic flow direction is obvious.

The time-of-day control should focus not only on the stability and orderliness of traffic flow, but also on the change in the instantaneous state of the main direction of internal flow. Therefore, classifying the peak period of intersections as a signal control period is unreasonable; doing so will lead to significant and unnecessary traffic delays and accident risks because the segmented point division design is poorly matched to the actual traffic conditions.

To sum up, the traditional total flow division method disregards two aspects. Firstly, simple judgment of intersection traffic is given priority, whereas important internal conversion in the direction of change is disregarded. The intersection traffic flow scheme for dividing the subsection point and traffic flow does not match the actual supply capability. Secondly, a segmentation point division model is used to solve the applicability of different types of intersections. The question we now seek to address is, “To which specific traffic intersection characteristics shall the model apply?” 

## 2. Literature Review

Most urban intersections in China are divided based on artificial experience, i.e., traffic engineering technicians draw the flow time curve according to the traffic flow of the intersection and manually classify the time-of-day control plan of the intersection by combining the control characteristics of the curve [5,6,7,8]. The traditional artificial classification method is mainly based on the engineering personnel’s judgment. The results are thus subjective and one-sided, and it is difficult to cope with the randomness of a new generation of urban traffic and sudden demand. To solve the limitations of the traditional artificial division method based on experience, Hauser and Scherer developed data mining tools by using SAS language on the basis of the traffic flow history data throughout the day. The distance and trends of each segment data and the centre cluster were judged on the basis of system state theory. The hierarchical clustering algorithm (HCA) was also used. Single-factor simple cluster analysis can be divided into several control periods. However, the HCA process is relatively simple, and an anomalous central cluster often forms during the process of data processing, which is distant from most of the central clusters and affects the clustering effect, commonly known as the dirty central cluster [9]. Park and Lee performed a single-factor judgment based on the traffic flow at an intersection for an entire day to further detail the maximum, minimum, and average values of traffic flow. A genetic algorithm (GA) was introduced to optimise the automatic partitioning of time-of-day segmented points; this effectively eliminated the problem of the dirty central cluster, which is easily generated by HCA, and obtained good operational results [10]. Yang et al. took the traffic delays at the intersection as the evaluation indices and compared and analysed them with the K-means algorithm based on mass historical flow data at the intersections through a Kohonen clustering algorithm, which proved the excellent effect of the K-means algorithm in clustering analysis. However, the K-means algorithm needed to set the clustering number and the initial clustering centre in advance, which could easily lead to local optima [11]. Ratrout sorted the traffic flow data of the intersection every 15 min according to the time sequence. The cluster number was determined by the Z-score indicators of intersection traffic flow and time variable. The method of combining a K-means algorithm was proposed to find the optimal segmentation point. Synchro simulation software was used to verify the effectiveness of the algorithm, but had no anti-interference capability against noise [12]. Chen and Yao determined the number of segmented points and the corresponding optimal switching time on the basis of traffic data repair and through the hybrid clustering algorithm. Initial clustering of historical traffic flow data was conducted by using the fast convergence of the K-means algorithm to improve the cubic group criterion as the clustering termination condition. The system clustering method was used for analysis and detailed clustering. The timing of the traffic data, added to the algorithm, was considered to reduce the disturbance of traffic state from the frequent switching of the segmented point scheme. The singular points in the segmented points were processed, which exerted certain effects [13,14,15,16,17]. 

The single-factor time division method has made considerable progress but still cannot meet actual demand. Therefore, multi-factor cluster analysis of traffic flow, delay, and stops was conducted by Abbas and Sharma [18]. The degree of detachment (DOD) was taken as the major evidence of a cluster. A high DOD indicated numerous segmented points. If the DOD was zero, then an intelligent traffic signal control scheme for an entire day was satisfactory. The TOD model was optimised by the algorithm of non-dominated sorting GA (NSGAII). The minimum sum of traffic delays and stopping times was taken as the objective function, and the Pareto front generated by the NSGAII algorithm was the segmented point of the time-of-day control scheme, which provided a new way of studying the multi-factor time period division method. Wang divided the total traffic flow at the intersections of four entrance directions (east, south, west, and north) of traffic diversion. The data smoothing process before the application of multi-factor cluster algorithm could prevent excessive noise. The model overcame the limitations of time-of-day control at a single intersection and was extended to the group of continuous multiple intersection times to coordinate control of the application [19,20,21,22,23]. In the application of the continuous intersection time-of-day control scheme, Park and Li took the signal cycle in the intersection signal control scheme as the feature vector to replace the intersection traffic flow as the main judgment factor of the multi-factor time period division. In the peak traffic oscillation area, greedy search algorithm and other heuristic algorithms, such as genetic algorithm, were compared and evaluated. The results showed that the greedy search algorithm performed better than other algorithms [24,25,26,27]. Lee used the multi-factor division of traffic flow and signal cycle to coordinate and control multiple intersections. Transportation cost was proposed as an important factor of the evaluation index. Unlike previous studies, which only considered the cost of transportation between segments, this study fully considered the transport costs during the transition period between two different control schemes [28,29,30,31,32]. An advanced cluster analysis method was applied in the coordinated control system, and a semi-actuated system was proposed by Guo. On the basis of a large number of real-time data offered by traffic data centres, a certain effect was obtained at the time of traffic as the main factor of time division, with the average speed of traffic delays, evaluation index, control of intersection, traffic signal timing plan cycle, and phase difference for time division multiple factors, such as clustering analysis [3,33,34,35,36,37,38,39,40,41].

In the above studies, parameters such as cycle and green letter ratio in the single-factor time division model require abundant and complicated calculations. The most important part of traffic control management is to observe the instantaneous change in internal flow of traffic stability. However, when the total flow is stable, single-factor division cannot accurately detect the flow differences between two major conflicts and cannot control them efficiently. In the multi-factor segmented division method, the problem of a singular object being extracted by a single factor is overcome. However, many influencing factors exist in the algorithm, which not only results in computational complexity, but also causes the time division to be too trivial, thereby seriously affecting control efficiency. Most studies on multi-period control segmented point division have mainly focused on the dimension of division factors. However, the data-driven and traffic safety method is used to refine the classification depth, and the adaptability analysis of different types of intersections remains at an initial stage.

## 3. Methodology

Along with the gradual improvement in traffic big data perception and deep excavation analysis technology, the data-driven approach provides numerous new ideas for traditional model construction and optimization analysis. Firstly, new dimensions and attributes are assigned to traditional data. The rapid development of traffic data perception technology has allowed the incorporation of several traffic elements around intersections into the big-data perception system, thereby increasing the data variety. Many attributes are given to traditional data through the analysis of the processing and relevance of mass data, thereby increasing the dimension of data. Secondly, the model construction presents exclusivity. The traditional model construction is based on an adjustment of the classical theoretical model and optimization of the correlative core control parameters to realise an analysis model that meets the majority of traffic characteristics. However, the big data model aims to break the shackles of traditional model construction, propose a matching model from the aspect of pure data processing analysis, and elaborately analyse the model applicability. The new model is likely to be applied only to the depth of a particular traffic characteristic, which is inapplicable to most situations but can play a significant role under certain conditions. In this study, the traditional time division model is optimised in two aspects on the basis of the input data in the data-driven method and the adaptive refinement of the model.

### 3.1. Procedural Framework

**Figure 2:**
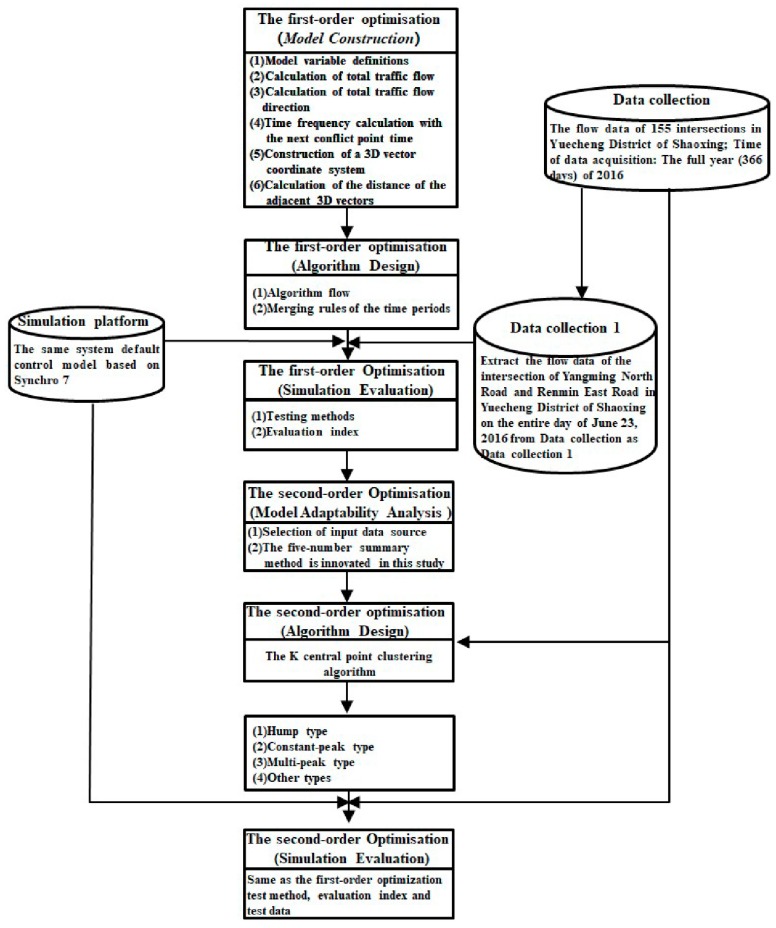
Procedural framework.

The procedural framework of this paper is shown in Figure 2.

### 3.2. First-Order Optimization (Model Construction of 3D Vector Time Division of Traffic Flow)

A model of 3D vector time division of traffic flow is established in this study. The model includes three factors, namely, the total amount of traffic flow at the intersection within 15 min, the total traffic flow direction, and the length of time between the data collection point and the next conflict point. 

#### 3.2.1. Model Variable Definitions

**Figure 3:**
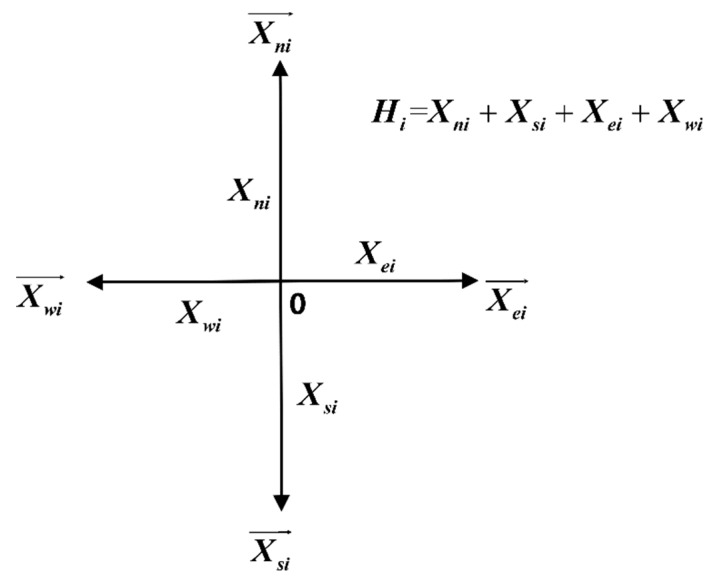
Variable definition diagram.

The total traffic flow of intersection is divided into four directions of traffic flow according to the principle of traffic flow in and out of an intersection. The variables $X_{e},X_{w},X_{s},X_{n}$ are defined to represent the import traffic flow in four directions (east, west, south, and north) at the intersection, and they have positive values. Data are collected every 15 min. Therefore, 96 traffic flow data points exist for a single intersection. The variable definition diagram is shown in Figure 3.

$T_{i}$ represents the ith time duration, i=1,2,3,⋯96.

$\vec{X_{ni}}$ represents the north traffic flow $\vec{X_{ni}}=\left(0,X_{ni}\right)$ at this time duration $T_{i}$. 

$\vec{X_{si}}$ represents the south traffic flow $\vec{X_{si}}=\left(0,X_{si}\right)$ at this time duration $T_{i}$. 

$\vec{X_{ei}}$ represents the east traffic flow $T_{i}$ at this time duration $\vec{X_{ei}}=\left(0,X_{ei}\right)$. 

$\vec{X_{wi}}$ represents the west traffic flow $T_{i}$ at this time duration $\vec{X_{wi}}=\left(0,X_{wi}\right)$.

#### 3.2.2. Calculation of Total Traffic Flow

In Figure 3, the total traffic flow $H_{i}$ represents the total flow of the four directions in the period $T_{i}$.

(1) $$H_{i}=X_{ni}+X_{si}+X_{ei}+X_{wi}$$

#### 3.2.3. Calculation of Total Traffic Flow Direction

Definition of total traffic flow direction: The intersection angle $\theta_{i}$ between the total vector $\vec{O_{i}}$ and the right horizontal axis intersection of the intersection of four inlet directions at the intersection of the time period $T_{i}$.

Step 1: Calculation of the fitting vector $\vec{Y_{i}}$ of traffic flow in the east and west entrances (horizontal axis): In this time period, $T_{i}$ east entrance traffic flow vector $\vec{X_{ei}}$—west entrance traffic flow vector $\vec{X_{wi}}$ is $\vec{Y_{i}}=\left(0,X_{ei}-X_{wi}\right)$.

Step 2: Calculation of the fitting vector $\vec{Z_{i}}$ of traffic flow in the south and north entrances (vertical axis): In this time period, $T_{i}$ north entrance traffic flow vector $\vec{X_{ni}}$—south entrance traffic flow vector $\vec{X_{si}}$ is $\vec{Z_{i}}=\left(X_{ni}-X_{si},0\right)$.

Step 3: In this time period, $T_{i}$ vector calculation of traffic subflow final fitting of the four-entrance direction $\vec{O_{i}}$ is

(2) $$\vec{O_{i}}=\vec{Y_{i}}+\vec{Z_{i}}=\left(\vec{X_{ei}}-\vec{X_{wi}}\right)+\left(\vec{X_{ni}}-\vec{X_{si}}\right)=\left(X_{ei}-X_{wi},X_{ni}-X_{si}\right).$$

Step 4: The angle calculation of the total vector of the ultimate fitting $\vec{O_{i}}$ with the intersection of the horizontal axis at the right side of the origin $\theta_{i}$ is

(3) $$\theta_{i}=\arctan\vec{O_{i}}=\arctan\left(\vec{X_{ei}}-\vec{X_{wi}}+\vec{X_{ni}}-\vec{X_{si}}\right)=\arctan\frac{X_{ni}-X_{si}}{X_{ei}-X_{wi}}.$$

**Figure 4:**
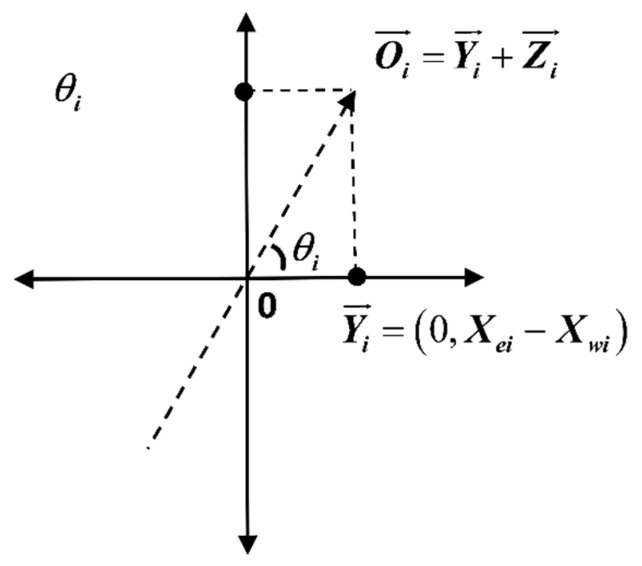
Diagram of the definition of total traffic flow direction $\theta_{i}$.

The diagram of the definition of total traffic flow direction $\theta_{i}$ is shown in Figure 4.

#### 3.2.4. The Calculation of the Traffic Safety Factor (Time Frequency Calculation with the Next Conflict Point Time)

$T_{k}$ represents the kth time duration, k=2,3,4⋯95.

(4) $$\left\{ \begin{array}{l} X_{n\left(k-1\right)}+X_{S\left(k-1\right)}\leq X_{e\left(k-1\right)}+X_{w\left(k-1\right)}\\ X_{nk}+X_{sk}>X_{ek}+X_{wk} \end{array}\right.\;or\;\left\{ \begin{array}{l} X_{n\left(k-1\right)}+X_{s\left(k-1\right)}\geq X_{e\left(k-1\right)}+X_{w\left(k-1\right)}\\ X_{nk}+X_{sk}<X_{ek}+X_{wk} \end{array}\right.$$

If any one of the two conditions in Equation (4) is satisfied, then $T_{k}$ is defined as the conflict point time. The time frequency calculation of this time $T_{i}$ duration and the next conflict point time $S_{i-k}$ is

(5) $$S_{i-k}=k-i,i<k.$$

#### 3.2.5. Construction of a 3D Vector Coordinate System

Definition of $\vec{\beta_{i}}$ 3D vector: The form of a 3D vector is used to represent the size, direction, and time frequency with the conflict point of the total traffic flow at a certain intersection in a certain time period. The 3D vector includes three factors, namely, (1) the total flow $H_{i}$ within time duration $T_{i}$, (2) the total traffic flow direction $\theta_{i}$ within time duration $T_{i}$, and (3) the time frequency with the conflict point $S_{i-k}$, and its specific representation is as follows:

(6) $$\vec{\beta_{i}}=\left(H_{i},\theta_{i},S_{i-k}\right).$$

$H_{i}$ can be calculated from Equation (1), $\theta_{i}$ can be calculated from Equations (2) and (3), and $S_{i-k}$ can be calculated from Equations (4) and (5).

#### 3.2.6. Calculation of the Distance of the Adjacent 3D Vectors

The distance of the adjacent 3D vectors under the 3D coordinate defined by this study is $m_{i}$. The starting point of all 3D vectors is the origin of the coordinate axis. Thus, the distance between adjacent vectors is calculated as the distance between the endpoints of the two vectors in the 3D space, as follows:

(7) $$m_{i}=\sqrt{{\left(H_{i}-H_{i+1}\right)}^{2}+{\left(\theta_{i}-\theta_{i+1}\right)}^{2}+{\left(S_{\left(i+1\right)-k}\right)}^{2}}.$$

The conflict point frequency of the adjacent two vectors is a fixed value, which cannot accurately describe the distribution of the conflict points inside the traffic flow. Therefore, the specific values of the time frequency of the vector distance conflict point in the adjacent vectors are taken from the adjacent vectors. The distance amongst the adjacent traffic flows of 3D vector $m_{i}$ considers the traditional model of traffic flow numerical size, and the directivity of traffic flow and the distribution of internal impulse points are also included.

### 3.3. First-Order Optimization Algorithm Design

Cumulative sum (CUSUM) time-series partitioning algorithm based on big data: The original flow sequence is divided into two subsequences with obvious physical significance, i.e., the latter part of the flow has a turning point relative to the previous one. The strategies of recursion and divide and conquer are used to process each subsection of the partition by subsection point until the length of the last subsequence is less than the minimum segment length threshold λ. The iteration can be terminated. Thus, a series of subsequences of unequal parts is obtained.

#### 3.3.1. Algorithm Flow

**Figure 5:**
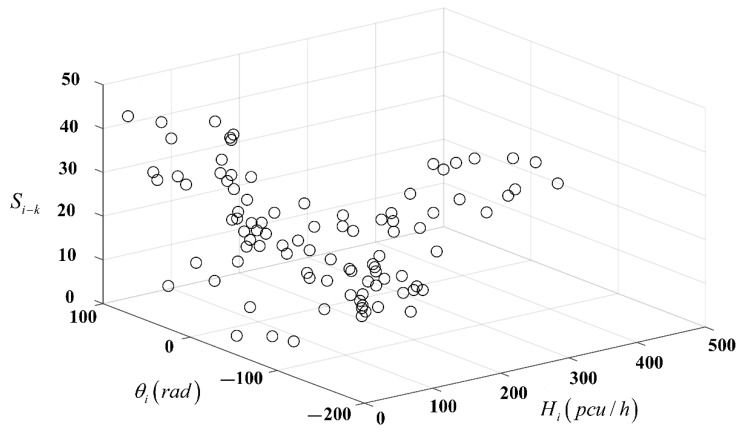
Traffic flow and vector distribution diagram of the entire day (Intersection at Yangming North Road and Renmin East Road on 23 June 2016 on the basis of MATLAB).

**Figure 6:**
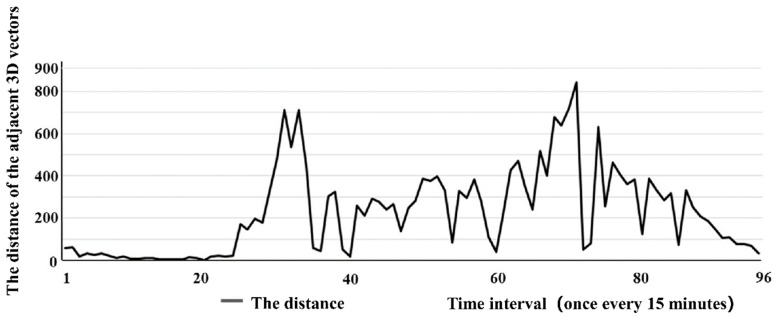
Space distribution chart of the adjacent 3D vectors (entire-day traffic flow at the intersection of Yangming North Road and Renmin East Road in Shaoxing City on 23 June 2016).

Ninety-six 3D vectors throughout the day are counted, i.e., the distance between the 95 adjacent 3D vectors $m_{i}$. As shown in Figure 5 and Figure 6, the average value of the distance amongst the three-dimensional vectors of adjacent traffic flow $m_{i}$ is calculated as

(8) $$\bar{m}=\frac{\sum_{i=0}^{k}m_{i}}{k+1},k=94.$$

where i represents the number of distance amongst adjacent traffic flows and vectors of the entire day $m_{i}$. The accumulation of each inner point of the sequence is calculated $m_{i}^{\prime }$ as

(9) $$m_{i}^{\prime }=0,\;i=0$$

(10) $$m_{i}^{\prime }=m_{i-1}^{\prime }+\left(m_{i}-\bar{m}\right),i=0,1,2,\ldots k.$$

When $\left|m_{p}^{\prime }\right|=\max\left\{\left|m_{i}^{\prime }\right|,i=0,1,2,\ldots k,k=94\right\}$, the corresponding time period $T_{i}$ is marked as a segmentation point, and the segmentation point sets are recorded as V. Time-of-day control requires certain stability and continuity, and excessive subsections can lead to overcomplicated control schemes and the ordering of normal traffic flow. Therefore, all the segmentation points in V need to be consolidated and optimised, and the specific regulations are as follows:

#### 3.3.2. Merging Rules of the Time Periods

Step 1: The shortest cut sequence is determined. If the time period width is less than or equal to $t_{i}=30\;\min$, then step 2 is performed; otherwise, the procedure is terminated. 

Step 2: The width of the adjacent time period in this time period is analysed. If the width of the time period is less than or equal to $t_{j}=30\;\min$, then it is merged with the shorter time period (if the time period is at the end of the boundary of the entire time series, then it merges directly with adjacent time periods), and step 1 is repeated; otherwise, step 3 is performed. 

Step 3: The mean values of the total traffic flow and the traffic direction flow in the time period are analysed and compared with those of the adjacent time periods. If $|\left|H_{i+1}\right|-\left|H_{i}\right||<\sigma$ and $\left|\theta_{i+1}-\theta_{i}\right|<\mu$, then the two time periods of $T_{i+1}$, $T_{i}$ are merged; otherwise, step 1 is repeated. (If the time width bin the segmented time periods is less than or equal to $t_{i}=30\;\min$, then merging shall be considered.) 

### 3.4. Second-Order Optimization (Model Adaptability Analysis of the Segmented Division Driven by Data)

With the explosive development of big data technology and the increasingly sophisticated and proprietary model constructed by data driving, the constructed model may be applied to the depth application under a specific traffic characteristic condition, which is inapplicable to most cases but will have a significant optimal control effect in a particular case. Therefore, this study uses big data clustering analysis to combine and classify all intersections, as indicated in Section 4. The adaptability of 3D vector time division methods to the intersection of various traffic flow characteristics is studied, and the most suitable intersection type of the method is determined. 

#### 3.4.1. Selection of Input Data Source

##### Five-Number Summary

Five-number summary is used to describe the global characteristics of a dataset. The immediate mastery of the data extremum and volatility change is the main statistical analysis method for analysing sample characteristics. The specific algorithm proceeds as follows [42,43]:

Step 1: The data are arranged in ascending order.
Step 2: The minimum value is confirmed.
Step 3: The first quartile (Q1) is confirmed.
Step 4: The median (Q2) is confirmed.
Step 5: The third quartile (Q3) is confirmed.
Step 6: The maximum value is confirmed.

##### A Five-Number Summary Method is Developed in this Study

The innovation of the time division model focuses on the difference in the direction of traffic flow in the near-peak buffer period (defining the peak period as the peak buffer period) and the distribution of impulse points. Therefore, the five-number summary method focuses not only on the high-peak and low-peak distribution of the macro traffic flow but also on the fluctuation in the peak buffer period. The specific algorithm is as follows:

Step 1: The data are arranged in ascending order.
Step 2: Five-number summary method is used to confirm five decision datasets.
Step 3: In accordance with the five decision datasets in step 2, the data are initially divided into several peaks and a flat hump. The peak period is defined as the peak buffer period.
Step 4: The average value in the peak buffer period is calculated.
Step 5: The variance in the peak buffer period is calculated.
Step 6: The maximum value in the peak buffer period is calculated.
Step 7: The distance between the adjacent conflict points in the peak buffer period is calculated.

Five fields are included in each core data namely, median (Q2), the average value in the peak buffer period, the variance in the peak buffer period, the maximum value in the peak buffer period, and the distance between the adjacent conflict points in the peak buffer period. 

#### 3.4.2. Second-Order Optimization Algorithm Design

Basic process of the central point clustering algorithm: Each cluster is optionally assigned a representative object, and the remaining objects are allocated according to their distance from each representative object to the cluster represented by the nearest representative object. Non-representative objects are repeatedly used to replace the representative objects to optimise the clustering quality. The clustering quality is represented by a cost function. When a central point is replaced with a non-central point, the remaining points are redistributed, except for the central points that are not replaced. The algorithm proceeds as follows [44,45,46]:

Step 1: K observed values are randomly selected (each value is called a central point).
Step 2: The distance/diversity of the observed value to each centre is calculated.
Step 3: Each observed value is assigned to the nearest central point.
Step 4: The sum of the distance from each of the central point to each observed value (total cost) is calculated.
Step 5: A point in the class that is not the centre is selected and swapped with the centre point.
Step 6: Each observed value is assigned to the nearest central point.
Step 7: The total cost is recalculated.
Step 8: If the total cost is less than the total cost calculated in step 4, then a new point is taken as the central point.
Step 9: Steps 5 to 8 are repeated until the central point does not change.

## 4. Case Study

### 4.1. Data Collection

Range of data acquisition: 155 intersections in Yuecheng District of Shaoxing. Time of data acquisition: the whole year (366 days) of 2016. Data volume: 5,446,080 pieces (96 pieces/day/item*155*366 days). Single data pattern: total traffic flow of each intersection within 15 min (unit: PCU/h) (a total of 96 pieces of data per day at each intersection). 

### 4.2. First-Order Optimization Simulation Evaluation

#### 4.2.1. Testing Data

As indicated in Section 4.1, the traffic flow at the intersection of Yangming North Road and Renmin East Road in Yuecheng District of Shaoxing for the whole day of 23 June 2016 is tested. The 3D vector of traffic flow at the intersection in 3D coordinates is calculated by the model in Section 3.2 and displayed based on MATLAB, as shown in Figure 5. A data point represents the end point of the 3D vector of the traffic flow within this time period $T_{i}$, and the starting point is the original point. The data point corresponds to the total traffic flow size $H_{i}$ (pcu/h) within this time period $T_{i}$, the value corresponding to the y-axis represents the total traffic flow direction $\theta_{i}$ (rad) within this time period $T_{i}$ and the value corresponding to the z-axis represents the time sequence of the conflict point $S_{i-k}$. The sequence number on each data point represents the sequence of the time period of data acquisition (i=1,2,3,⋯96). Equation (7) is used to calculate the distance between adjacent 3D vectors, as shown in Figure 6. 

#### 4.2.2. Simulation Platform and Testing Methods

The traditional method also adopts CUSUM to ensure the objectivity of the comparison analysis of the model, but the input data sources are different. The input sources in the innovated method replace the traditional total flow with 3D vectors.

#### 4.2.3. Evaluation Index

Total delay time of the entire day (its calculation formula is the sum of the waiting time for all vehicles passing through this intersection) (unit: h).

**Figure 7:**
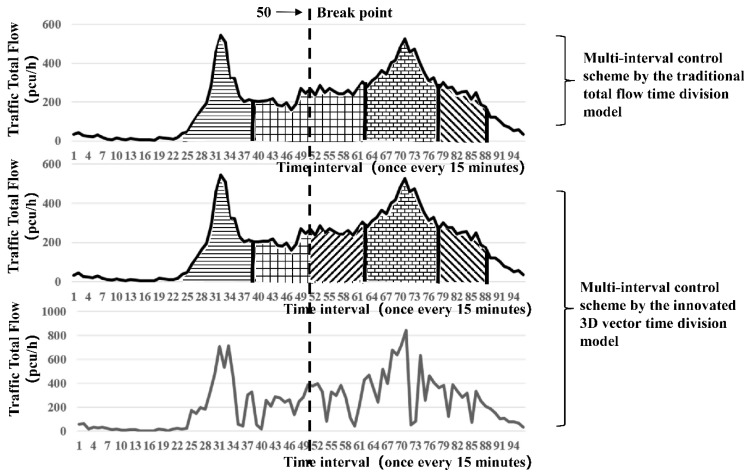
Scheme comparison diagram of the 3D vector time period division with the traditional total flow division (entire-day traffic flow at the intersection of Yangming North Road and Renmin East Road in Shaoxing on 23 June 2016).

The results obtained through the simulation are shown in Figure 7 and Table 1 and Table 2.

Figure 7 shows that the time-of-day control scheme at the intersection is divided into four time periods by the traditional total flow time period division model, whereas the 3D time period model innovated in this study divides it into five time periods. The above two methods have roughly the same time periods. The only difference is that the innovated method under the traditional method of serial numbers 40 to 63 interval period for 10 points to 15 points in the conversion (time: 45 points) is divided into two periods (serial number: 50 piecewise points), which is more sophisticated. The innovated method considers not only the intersection supply matching capacity of the total flow but also the fundamental transformation of traffic flow direction during the period.

A comparison of the above two tables indicates that the total delay time in the entire day obtained by the traditional total flow method is 74 h, whereas that of the polar coordinate time division method is 68.5 h. Compared with traditional total flow time period division, 3D time period division reduces 5.5 h of the entire-day total delay time, i.e., approximately 7.4%. Especially during the time period near the peak from 10:00 to 15:45, the total delay time under the innovated method is 7.25 + 10 = 17.25 h, whereas that of the traditional total flow method is 22.75 h. Unlike the traditional method, the innovated method reduces the delay time by 5.5 h, which is approximately 24% throughout the day.

### 4.3. Second-Order Optimization Simulation Evaluation

#### 4.3.1. Testing Data

The 5.4 million basic data points from Section 4.1 are filtered and extracted using the five-number summary method in Section 3.4, and 56,730 core data points are obtained as input data sources for the following large data clustering analysis. 

#### 4.3.2. Data Clustering Analysis Simulation Evaluation

Simulation platform: R-Studio; algorithm: K central point clustering algorithm.

**Figure 8:**
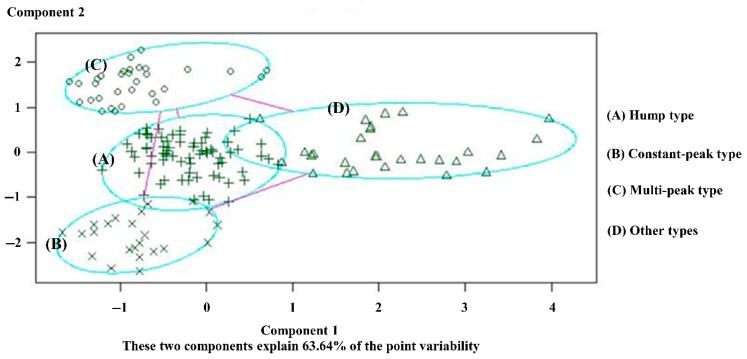
Simulation diagram of K central point clustering algorithm.

**Figure 9:**
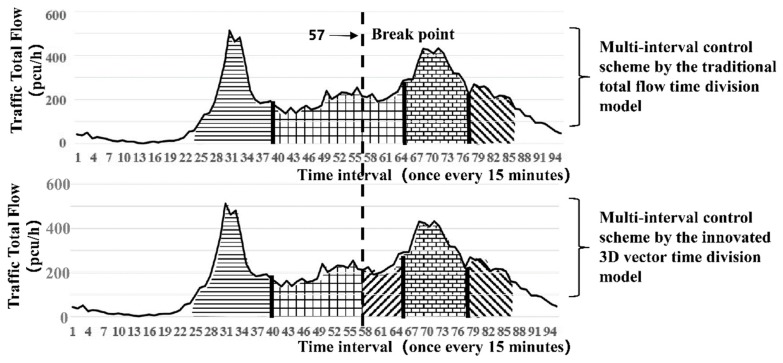
Scheme comparison diagram of the 3D vector time period division with the traditional total flow division (hump-type representative: No. 6 intersection of Jiefang South Road and South Ring Road).

**Figure 10:**
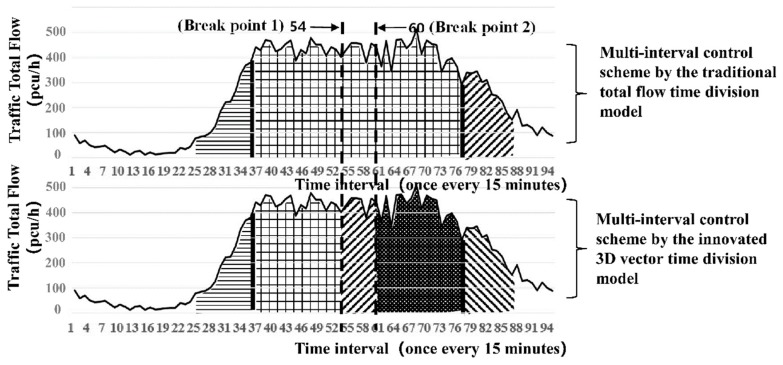
Scheme comparison diagram of the 3D vector time period division with the traditional total flow division (constant-peak-type representative: No. 79 intersection at Pingjiang Road and Renmin East Road).

**Figure 11:**
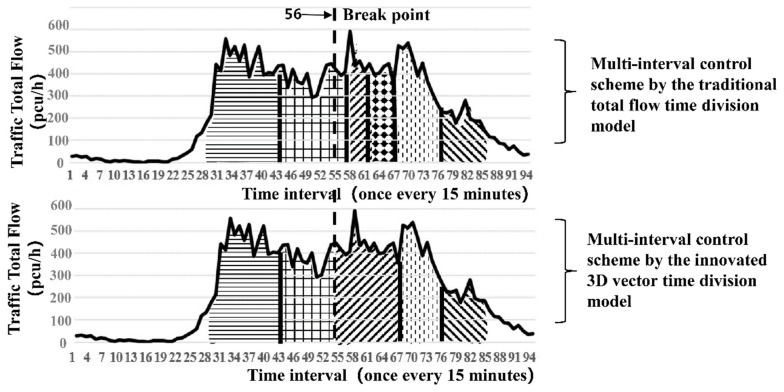
Scheme comparison diagram of the innovated time period division with the traditional total flow division (multi-peak-type representative: No. 61 intersection at Jiefang North Road and Renmin West Road).

Through simulation, the big data clustering algorithm is used to divide the 155 intersections of Yuecheng District in Shaoxing City into four categories according to different traffic flow characteristics, as shown in Figure 8.

(A) Hump type: High-peak and low-peak hours in the morning and evening exist in the total flow, and the concussion amplitude is large. The curved shape is similar to a camel’s hump. The distribution of the inside conflict point is comparatively uniform. For example, intersection no. 8 (Jiefang South Road and South Ring Road) is illustrated in Figure 9. 

(B) Constant-peak type: This type is characterised by a large total flow, which is mostly in shock peak and normal peak flow. Low peak hardly exists or occurs for an extremely short time. For example, for intersection no. 79 (Pingjiang Road and Renmin East Road), many inside conflict point exists throughout the period, the details are shown in Figure 10.

(C) Multi-peak type: Many false peaks exist between the morning and evening peaks. The distance between the false peaks is relatively dispersed. The total traffic volume varies with the conflict point; thus, identifying the turning point of the time division is easy. For example, intersection no. 61 (Jiefang North Road and Renmin West Road) is presented in Figure 11. 

(D) Other types: No obvious rule for the peaks exists, and the internal conflict points are varied and chaotic. Therefore, the innovated method cannot be used to identify the segmented points. This paper only analyses the adaptability of the three intersection types above. 

The location table of the three types of intersection clustering central point is shown in Table 3.

#### 4.3.3. Adaptability Analysis

(1) Testing objective: To test the adaptability of the innovated method of time division into four intersection types
(2) Testing data: Intersection data of the four types (155 in total)
(3) Testing method: First-order optimization simulation evaluation
(4) Evaluation index: Total delay time for the entire day (its calculation formula is the total time spent by cars waiting at the intersection) (unit: h)
(5) Testing platform: The same system default control model based on Synchro 7

A typical intersection is taken as a display in each category of the four types of intersections, and the results are shown in Figure 9, Figure 10 and Figure 11 and Table 4.

Table 4 shows that the traditional total flow division method has a lower traffic delay than the three types of intersections. The effect of time division is especially obvious for the hump-type intersection, in which the average daily total delay is reduced by approximately 5.6% (5.14 h), and the average daily peak buffer time delay is reduced by approximately 17%. For the constant-peak and multi-peak types, the delay increases slightly. Thus, the innovation method is highly exclusive. Its control effect needs to be improved for large and persistent traffic flow, especially for intersections with numerous disorderly internal transition points in the high-peak buffer period. 

## 5. Conclusions

A double-order optimization model of time-of-day control segmented point division is developed in this study, and the traditional model is optimised step by step. First-order optimization (deep optimization of the model input data) gives the traffic flow directivity, increases the dimensions of the traditional traffic flow data, and reconstructs and optimises the classic model. The form of the traffic safety factor 3D vector is used to represent the size, direction, and distribution of the conflict point of the total traffic flow at a certain intersection in a certain time period. CUSUM, a time series segmentation algorithm, is used to recurse and merge the distance amongst adjacent vectors to confirm each of the segmented point of the time-of-day control scheme. In the second-order optimization (analysis of model adaptability), the actual traffic flow data of 155 intersections in Yuecheng District of Shaoxing are adopted as the testing data and processed using the five-number summary method. The processed data are analysed with big data K central point clustering and divided into four intersection types, namely, hump-type (obvious morning and evening peaks and low peaks occur, the oscillation amplitude between high and low peaks is large, and the distribution of the internal conflict points is uniform), constant-peak type (large total flow mostly oscillating around the high peaks and a constant high-peak flow), multi-peak type (several false peaks exist during the morning and evening high peaks, and the false peaks are widely dispersed), and other types (no obvious rules exist in the high and low peaks, and many chaotic internal conflict points exist). The four intersection types in the tested range are evaluated separately by the innovated segmented division model and the traditional single-factor total traffic flow segmentation division model on the basis of Synchro 7 simulation software. The results show that when the innovated double-order optimization model is used in the intersection according to the hump-type traffic flow characteristics, its control is more accurate and efficient than that of the traditional total flow segmentation model, which exerts a certain engineering implementation effect. For the constant-peak and multi-peak types, the delay increases slightly, thereby indicating that the exclusivity of the model is strong. The next focus of this study will be the traffic characteristics of the other three non-hump types to improve and optimise the model. The big data clustering analysis algorithm for intersections will also be analysed in depth to determine the potential rules for intersections of other types with weak regularities. 

## Figures and Tables

**Table 1:** Time-of-day division scheme under the traditional total flow time period division of the intersection at Yangming North Road and Renmin East Road.

Serial No. of Time Period	Starting and Ending Times	Duration of the Time Period (h)	Total Delay Time of the Entire Day (h)
A	6:00–10:00	4.0	12.35
B	10:00–15:45	5.75	22.75
C	15:45–19:45	4.0	24.5
D	19:45–22:00	2.15	7.25
E	22:00–6:00	8.0	7.15
Total	--	24	74.0

**Table 2:** Time-of-day division scheme under 3D vector time period division of the intersection at Yangming North Road and Renmin East Road.

Serial No. of Time Period	Starting and Ending Times	Duration of the Time Period (h)	Total Delay Time of the Entire Day (h)
A	6:00–10:00	4.0	12.35
B	10:00–12:30	2.5	7.25
C	12:30–15:45	3.25	10.0
D	15:45–19:45	4.0	24.5
E	19:45–22:00	2.15	7.25
F	22:00–6:00	8.0	7.15
Total	--	24	68.5

**Table 3:** Location table of the three types of intersection clustering central point.

No.	Intersection Types	Average Value in the Peak Buffer Period	Variance in the Peak Buffer Period	Maximum Value in the Peak Buffer Period	First Quartile (Q1)	Third Quartile (Q3)
1	Hump type	241.734	350.123	359	70	315
2	Constant-peak type	613.796	5173.218	695	50	579
3	Multi-peak model	728.532	2803.248	803	181	753

**Table 4:** Adaptability analysis table of the four types of intersections by the innovated time period division.

No.	Intersection Types	Average Daily Delay under the Traditional Total Flow Time Division Method (h)	Average Daily Delay under the Innovated Flow Time Division Method (h)	Average Daily Delay Time within the Peak Buffer Period under the Traditional Total Flow Period Division Method (h)	Average Daily Delay Time within the Peak Buffer Period under the Innovated Time Division Method (h)
1	Hump type	91.375	86.235	26.75	22.2
2	Constant peak type	29.75	32.61	23.78	27.03
3	Multi-peak type	25.43	26.87	13.817	14.653
Total		146.555	145.715	64.347	63.883
